# A Preliminary Study on Deep Learning-Based Plan Quality Prediction in Gamma Knife Radiosurgery for Brain Metastases

**DOI:** 10.3390/cancers17183056

**Published:** 2025-09-18

**Authors:** Runyu Jiang, Yuan Shao, Yingzi Liu, Chih-Wei Chang, Aubrey Zhang, Malvern Madondo, Mohammadamin Moradi, Aranee Sivananthan, Mark C. Korpics, Xiaofeng Yang, Zhen Tian

**Affiliations:** 1 Department of Radiation & Cellular Oncology, University of Chicago, Chicago, IL 60637, USA; 2 Department of Physics, University of Chicago, Chicago, IL 60637, USA; 3 Department of Radiation Oncology, Rush University Medical Center, Chicago, IL 60612, USA; 4 Department of Radiation Oncology, Emory University, Atlanta, GA 30322, USA

**Keywords:** Gamma Knife radiosurgery, brain metastases, plan quality prediction, deep learning, HD-U-Net

## Abstract

Background/Objectives: GK plan quality is strongly affected by lesion size and shape, and the same evaluation metrics may not be directly comparable across patients with different anatomies. This study proposes a deep learning-based method to predict achievable, clinically acceptable plan quality from patient-specific geometry. Methods: A hierarchically densely connected U-Net (HD-U-Net) was trained at the lesion level to predict 3D dose distributions for the estimation of plan quality metrics, including coverage, selectivity, gradient index (GI), and conformity index at a 50% prescription dose (CI50). To improve the prediction accuracy of plan quality metrics, Dice similarity coefficient losses for the 100% and 50% isodose lines were incorporated with conventional mean squared error (MSE) loss. Results: Ten-fold cross-validation on 463 brain metastases (BMs) from 175 patients showed that our method achieved smaller mean absolute errors across all four metrics than the HD-U-Net baseline trained with MSE loss. Improvements were pronounced in all metrics for small metastases, and were observed primarily in GI and CI50 for medium and large lesions. Paired Wilcoxon signed-rank tests confirmed the statistical significance of these improvements (*p* < 0.05). Conclusions: The proposed method outperformed the baseline model in capturing overall trends, improving per-lesion accuracy, and enhancing robustness to dataset variability. It can serve as a pre-planning tool to guide planners in constraint setting and priority tuning, a post-planning quality control tool to identify subpar plans that could be substantially improved, and as a foundation for developing deep reinforcement learning-based automated planning of GK treatments for brain metastases.

## Simple Summary

Gamma Knife (GK) radiosurgery is a precise, non-invasive treatment for brain metastases, with plan quality highly dependent on tumor size and shape. Although standard metrics are commonly used to assess plan quality, similar values may not correspond to the same quality level for patients with varying anatomies. As a result, GK treatment planning and plan evaluation often rely on planner and physician experience and trial and error, leading to substantial variation in plan quality across patients. In this study, we propose a deep learning-based approach that predicts the clinically optimal GK plan quality for each patient based on patient-specific anatomy. This tool can serve as a clinical quality control tool, identifying suboptimal plans, prompting further refinement, and ultimately reducing plan quality variation for future patients. In addition, it may support more consistent and automated treatment planning in the future.

## 1. Introduction

Gamma Knife (GK) radiosurgery is a specialized form of stereotactic radiotherapy for brain metastases (BMs) [1,2,3], delivering high radiation doses with sub-millimeter precision to eradicate tumors while achieving rapid dose fall-off to spare surrounding normal tissues. Individualized, high-quality treatment planning is critical to ensure treatment efficacy and patient safety. 

Traditional GK planning relies on manual forward planning, where planners determine the number and locations of isocenters, beam collimation, and beam-on time through a trial-and-error process. This process is cumbersome and complex due to the vast solution space, resulting in plan quality that varies with planners’ skills, experiences, and the effort devoted to planning [4,5]. Inverse planning was introduced to streamline the process and reduce variability by optimizing dose through a mathematic optimization problem with multiple objectives [6,7,8]. While it enables faster plan generation, manual tuning of objective priorities is still required, leaving final plan quality still partially dependent on planners’ experience [5,9]. Additionally, GK plan quality is strongly affected by the size and shape of treatment target, and commonly used plan quality metrics such as coverage, selectivity, and gradient index (GI) may not be comparable across patients with different anatomies. Consequently, physicians often rely on experience and trial-and-error to assess whether a plan can be improved. Plan quality control that considers patient-specific anatomy is therefore highly desirable for GK radiosurgery to identify plans with potential for substantial improvement and ensure consistently high quality across patients. 

Automated treatment planning offers an alternative and promising solution to reduce inter-planner variability [10,11]. In linac-based radiotherapy, deep learning-based automated planning has been explored primarily through two approaches. The first approach predicts a 3D dose distribution from prior patient data [12,13,14,15], which is then converted into a deliverable plan via dose mimicking [16,17]. However, this approach does not guarantee that the predicted dose is compliant with hardware constraints and physically deliverable. The second approach employs deep reinforcement learning (DRL) to learn decision-making in manual priority tuning during inverse planning [18,19,20,21,22]. While this DRL-based approach can ensure plan deliverability, its performance heavily relies on the design of an effective reward function [18,19,20,21], which in turn requires a clear definition of what constitutes high-quality plans. Recently, several studies have applied these deep learning-based automated planning approaches to GK radiosurgery. Zhang et al. employed generative adversarial networks (GANs) and U-Net architectures to predict dose distributions of GK treatment for BMs [23]. Their key finding was that lesion-level network training improves dose prediction accuracy compared with traditional patient-level training, due to large variability in BM number, size, and location across patients. They reported comparable performance between the lesion-level GAN and the lesion-level U-Net trained with conventional mean square error (MSE) loss, but U-Net required significantly less training time and was hence recommended as the more practical option for GK applications. This study also noted that accurate voxel-wise dose prediction is inherently challenging in GK radiosurgery due to variability in the number and placement of isocenters [23]. In their subsequent study on dose prediction-based automated GK planning for BM treatments [24], it was further found that the predicted dose distribution may not be physically deliverable by the GK systems; Attempting to precisely mimic the predicted dose distribution voxel-by-voxel often resulted in plans of lower quality than those manually generated by planners. Our group previously developed a DRL-based automated GK planning framework for vestibular schwannoma [9], where plan quality expectations are well defined due to relatively consistent tumor characteristics (e.g., location, shape). Extending this approach to BMs, however, is challenging because plan quality metric values of high-quality GK plans vary widely across BMs. Without a reliable method to estimate the optimal plan quality for each case, it is difficult to design an effective reward function for DRL-based automated planning for BMs. Therefore, accurate patient-specific plan quality prediction is also a critical prerequisite for enabling automated GK planning for BM treatment.

Motivated by these findings and challenges, this study shifts the focus from voxel-wise dose prediction accuracy to improving the accuracy of predicted plan quality metrics for GK radiosurgery of BMs. We adopted the lesion-level network training strategy proposed by Zhang et al. [23], for a hierarchically densely connected U-Net (HD-U-Net). This U-Net variant, originally developed by Nguyen et al. [13], combines the strengths of DenseNet [25] and U-Net [26] to capture both local and global information while maximizing information flow and computational efficiency. The HD-U-Net model has demonstrated success in predicting dose distributions for linac-based volumetric-modulated arc therapy in head and neck cancer [13]. Because GK plan quality metrics, such as coverage, selectivity, and gradient index (GI), are calculated based on the 100% and 50% isodose lines of the plan dose distribution, we designed an additional loss term that calculate the Dice similarity coefficients (DSC) of these two isodose lines between the predicted and ground truth dose distributions. This loss term is used alongside the conventional MSE loss for dose prediction network training to better enforce accurate prediction of GK plan quality. The following sections present our patient dataset, network architecture, training strategy, and evaluation metrics, followed by a detailed presentation of experimental results, a discussion of key findings and limitations, and final conclusions. 

## 2. Materials and Methods

### 2.1. Patient Dataset

With Institutional Review Board (IRB) approval, we collected treatment data from 175 patients with BMs who underwent GK radiosurgery at our collaborating institution. The dataset includes T1-weighted MRI images, physician-delineated contours that were used for treatment planning, and the dose distribution from clinically approved GK treatment plans.

Given the substantial variability in the number, size, shape, and location of BMs across patients, we adopted a lesion-level network training strategy, as in Zhang et al. [23], rather than the conventional patient-level approach used in dose prediction studies of primary cancers [12,13,14,15,16,17]. In this retrospective study, BMs located near critical organs or adjacent to other BMs were excluded because of their limited amount in our dataset and/or their distinct dose distribution characteristics arising from the need of considering organ sparing or overlapping doses from neighboring BMs. The exclusion criteria are defined as follows: (1) Near critical organs: Because cranial organs at risks (OARs) have varying dose tolerances and prescription doses for BMs also differ, a fixed distance threshold was not appropriate. In this study, a BM was considered near a critical organ if (a) the organ was fully or partially contained within the tumor space, and (b) the organ’s dose endpoint from the clinical plan exceeded 50% of its tolerance. This conservative criterion ensures that the dose distributions of the BMs included in our dataset were largely unaffected by OAR-sparing constraints. An exception was made for BMs located within the brainstem, where the prescription dose had already been reduced to match the brainstem tolerance, eliminating the need for additional dose shaping to spare healthy brainstem tissue; these cases were hence retained in the dataset; (2) Adjacent to other BMs: A BM was considered adjacent to another lesion if their dose distributions overlap such that selectivity and GI could not be calculated. In such cases, the commercial GK treatment planning system Leksell Gamma Plan^®^ (Elekta AB, Stockholm, Sweden) reported the affected metrics as unavailable. Therefore, such BMs were excluded to avoid dose interference and to ensure that the dose distribution of each BM included in our dataset could be analyzed independently. Based on these criteria, 10 BMs were excluded due to proximity to critical organs, 399 due to adjacency to other lesions, and 108 due to both reasons. The final dataset consisted of 463 BMs. 

Table 1 summarizes detailed information of our dataset, including BM volume, prescription dose, and plan quality metrics calculated from the dose distributions of collected clinically approved GK plans. The plan quality metrics include coverage, selectivity, GI, and conformity index at 50% prescription dose (CI50), which are defined as:

(1) $$\mathrm{Coverage}\;=\frac{TV\cap PIV}{TV},$$

(2) $$\mathrm{Selectivity}=\frac{TV\cap PIV}{PIV},$$

(3) $$GI=\frac{{PIV}_{{0.5R}_{x}}}{PIV},$$

(4) $$CI50=\frac{{PIV}_{{0.5R}_{x}}}{TV}$$

**Figure 1:**
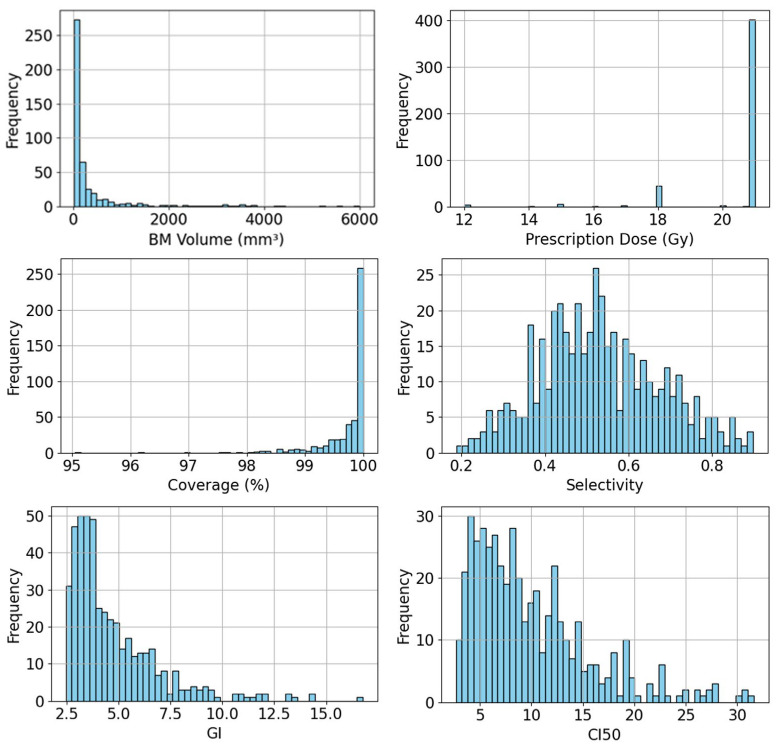
Histograms of BM volume, prescription dose, and plan quality metrics (e.g., coverage, selectivity, GI, CI50) of collected GK plans for the BMs included in our dataset.

Here, *TV* and *PIV* denote the target volume and the plan isodose volume receiving at least the prescription dose, respectively. ${PIV}_{{0.5R}_{x}}$ denotes the plan isodose volume receiving at least 50% of the prescription dose $R_{x}$. All four metrics are dimensionless and represent different volume ratios, although coverage is commonly expressed as a percentage in clinical practice. The first three metrics, coverage, selectivity, and GI, are standard metrics in GK planning to assess plan quality. CI50, also knowns as intermediate dose spillage, is widely used in linac-based stereotactic radiosurgery [27] and stereotactic body radiation therapy [28,29]. We included CI50 in our study as an additional quality metric for GK plans due to certain limitations of GI. While GI was designed to differentiate between plans with similar conformity but different dose gradients, primarily for evaluating normal tissue sparing [30], it becomes less suitable when comparing plans with different conformity [31]. In contrast, CI50 also considers ${PIV}_{{0.5R}_{x}}$ but normalizes this volume by *TV* rather than PIV, removing dependence on conformity and allowing direct comparison of normal tissue sparing among plans with varying conformity levels. Figure 1 presents the histograms of BM volume, prescription dose, and each plan quality metric of collected GK plans in our dataset, illustrating both the diversity of BMs in the dataset and the variability in plan quality metrics among clinically approved GK plans.

### 2.2. Data Preparation

To prepare the collected patient data for lesion-level training, we adopted the concept of tumor space from Zhang et al. [23], where each tumor space was originally defined as the smallest bounding box enclosing a BM at the center with a 1 cm margin. However, we found that this fixed margin was insufficient to capture an adequate surrounding dose region for large BMs. To address this, we redefined the tumor space as the 30% isodose volume of the prescription dose for each BM. There are several reasons for this choice. First, accurate computation of plan quality metrics requires dose data beyond the target: coverage and selectivity depend on the 100% isodose volume, while GI and CI50 also require the 50% isodose volume. Thus, the tumor space for each BM must extend at least to the 50% isodose volume to provide adequate context for plan quality prediction. Second, using a lower isodose threshold would capture a larger dose region but substantially increase data size, requiring a larger network architecture with higher computational cost. Third, given that the highest prescription dose in GK BM treatments is typically up to 25 Gy, 30% of this value remains below the lowest cranial OAR tolerance (8 Gy D_2cc_ for the optical pathway), ensuring that the tumor space is clinically safe to use. Thus, our definition of the tumor space as the 30% isodose volume represents a practical balance between including a sufficient dose region and maintaining computational efficiency. 

Each tumor space consisted of two types of data. The first was a 3D mask, used as the network input. It was centered on the BM, with voxel values assigned as 2 for the contoured BM volume, 1 for the patient skull, and 0 for voxels outside the body. Including skull information enabled the network to distinguish superficial lesions from deeply seated ones, which exhibit different dose distribution patterns. The mask was generated from physician-delineated contours. The second data type was the ground truth 3D dose distribution within the tumor space, extracted from the DICOM dose file of the corresponding clinical plan. Because prescription dose varies across BMs (due to differences in lesion size and prior treatments), all dose distributions were normalized by their respective prescription dose, enabling the network to learn consistent dose patterns across cases more effectively. 

To standardize dimensions across the dataset, the dose data was zero-padded outside the tumor space to a uniform size of 128 × 128 × 128 voxels at 0.5 mm isotropic resolution. The mask data was matched to this size but obtained differently. Rather than zero-padding, the entire 3D mask volume was directly truncated from the patient-level mask. Handling the mask data in this way ensures that the network can generalize to new cases without requiring the 30% isodose information, which is unavailable at the time of treatment planning. In addition, since BMs located near critical organs or adjacent to other BMs were excluded from training, any organ or neighboring BM contours within the 128 × 128 × 128 volume were ignored during mask generation. 

### 2.3. Network Architecture

**Figure 2:**
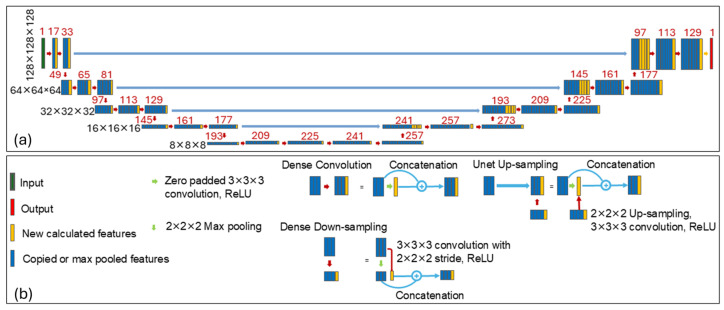
Network architecture of the HD-U-Net is shown in (**a**), with the detailed legend provided in (**b**). In (**a**), black numbers on the left denote the volume dimensions at each hierarchy level, while red numbers represent the number of feature maps at each layer. The 1-channel input (green block) undergoes a combination of operations of dense convolution, dense down-sampling, and U-Net up-sampling, followed by a final convolution layer with ReLU activation (yellow arrow). Orange blocks represent newly calculated features with trainable parameters, while blue blocks represent copied or max-pooled features that do not need trainable parameters. The network outputs the predicted 3D dose distribution (red block).

We employed a HD-U-Net architecture proposed by Nguyen et al. [13], which combines key principles of U-Net and DenseNet, to predict the optimal 3D dose distribution for each BM, enabling the estimation of optimal plan quality. As illustrated in Figure 2, the network consists of three types of operations: dense convolution, dense down-sampling, and U-Net up-sampling, arranged in a hierarchically and densely connected manner. Each dense operation incrementally adds a fixed number of new features based on a predefined growth rate, promoting feature reuse and efficient learning. A more detailed description of the network architecture can be found in Nguyen et al. [13]. In our implementation, we used a growth rate of 16 features (i.e., 16 new features added after each dense convolution or dense down-sampling operation), and 64 features returned during the U-Net up-sampling operation. 

### 2.4. Network Training

Zhang et al. reported that their dose prediction model performed worst near the target periphery due to sharp dose gradients there [23], which in turn affected the accuracy of plan quality metrics calculated from the predicted dose distribution. Since key metrics (e.g., coverage, selectivity, GI, and CI50) are derived from the 100% and 50% isodose lines of the plan dose distribution, in this study we introduced a DSC loss term for network training, alongside the conventional MSE loss, to enforce similarity between the isodose lines of the predicted and ground truth dose distributions for more accurate prediction of GK plan quality. The DSC loss is defined as:

(5) $$\mathrm{DSC}\;\mathrm{loss}\;=\frac{\sum_{i=1}^{N}{\left[\sigma \left(\frac{D_{pred}\left(i\right)}{R_{x}}-a_{iso}\right)-\sigma \left(\frac{D_{true}\left(i\right)}{R_{x}}-a_{iso}\right)\right]}^{\;2}}{\sum_{i=1}^{N}\left[\sigma {\left(\frac{D_{pred}\left(i\right)}{R_{x}}-a_{iso}\right)}^{2}+\sigma {\left(\frac{D_{true}\left(i\right)}{R_{x}}-a_{iso}\right)}^{2}\right]}\;,$$

where $\sigma \left(x\right)=\frac{1}{1+e^{-1000x}}$.

Here, $D_{pred}\left(i\right)$ and $D_{true}\left(i\right)$ denote the predicted and ground truth dose values for voxel i. $R_{x}\;$denotes the prescription dose, and $a_{iso}$ is set to 1.0 when calculating the DSC loss for the 100% isodose line and 0.5 for the 50% isodose line. N denotes the total number of voxels within the tumor space. The function $\sigma \left(x\right)$ is a modified sigmoid function as a smoothed approximation of a step function. In this study, we also modified the conventional MSE loss that is commonly used for dose prediction network training into a weighted MSE loss (referred to as wMSE), defined as:

(6) $$\mathrm{wMSE}\;\mathrm{loss}\;=\frac{1}{N}\sum_{i=1}^{N}\frac{{\omega_{i}\times \left|D_{pred}\left(i\right)-D_{true}\left(i\right)\right|}^{2}}{{R_{x}}^{\;2}},$$

allowing us to enforce varying levels of accuracy to different subregions. Here, $\omega_{i}$ is assigned heuristically to 8 for voxels receiving at least the prescription dose in the ground truth dose distribution, and 1 for all other voxels inside the tumor space. The total loss function used for training in our study is a weighted sum of the wMSE loss and the DSC losses for both isodose levels,

(7) $$Total\;loss=wMSE\;loss+\alpha \cdot DSC\;loss\left(a_{iso}=1\right)+\beta \cdot DSC\;loss\left(a_{iso}=0.5\right).$$

In our experiments, α and β were set to be 100 and 10, respectively, based on empirical hyperparameter tuning using a coarse logarithmic grid search. 

To mitigate overfitting, we performed on-the-fly data augmentation to enhance the diversity of the dataset. Our augmentation strategies included translations along the superior–inferior, left–right, anterior–posterior directions, rotations around the superior–interior axis, and left–right flips. Specifically, the rotational augmentation was applied using one of eight discrete angles (e.g., 0°, 45°, 90°, 135°, 180°, 225°, 270°, 315°), each selected with equal probability. These eight angle options reflect the 8-fold rotational symmetry of the radiation source sectors in GK systems, thus maintaining realistic dosimetric orientations. Each training sample had a 75% probability of undergoing augmentation, with equal chances of being translated or rotated. After translation or rotation, the left–right flip was applied with a 50% probability. Please note that no geometric deformations were applied during augmentation, as preserving the precise dose distribution of each tumor space is crucial for accurate prediction of plan quality metrics.

### 2.5. Evaluation

To assess the performance of our proposed method, we conducted ten-fold cross-validation. Specifically, the entire dataset of 463 BMs was randomly divided into ten subsets. In each fold, nine subsets were used for training and the remaining one for validation. The network was trained for 80 epochs per fold, with each epoch consisting of 200 batches and a batch size of 2. As a result, the training data of the nine subsets were approximately seen once per epoch. In our experiment, an exponentially decaying learning rate was employed, starting at 0.001 and decaying by a factor of 0.93 every 200 batches. A dropout rate of 10% was applied to mitigate overfitting.

To quantitatively evaluate the prediction accuracy of our method and compare it against a baseline HD-U-Net model trained with conventional MSE loss, we calculated the mean absolute error (MAE) for each predicted plan quality metric. Given that the values of several GK plan quality metrics (e.g., selectivity, GI, and CI50) highly depend on target volume, we further divided the dataset into four groups based on BM volume and evaluated prediction performance within each group. These groups were defined using the three available GK collimation sizes (4 mm, 8 mm, 16 mm in diameter), resulting in the following volume ranges: <33.5 mm^3^ (i.e., group 1 consisting of 71 BMs), 33.5–268.1 mm^3^ (group 2 consisting of 271 BMs), 268.1–2144.7 mm^3^ (group 3 consisting of 97 BMs), and ≥2144.7 mm^3^ (group 4 consisting of 24 BMs). To assess statistical significance, we conducted Paired Wilcoxon signed-rank tests comparing our proposed method and with the MSE method.

## 3. Results

This study was conducted on a workstation equipped with an Intel^®^ Xeon^®^ Gold 6258R CPU at 2.70GHz and a NVIDIA RTX A6000 GPU. The deep learning model was implemented and trained using TensorFlow. The training process of the ten-fold cross-validation took approximately three days in total using GPU. Once trained, the model took about 240 milliseconds to predict a 3D dose distribution to estimate plan quality metrics for each BM.

**Figure 3:**
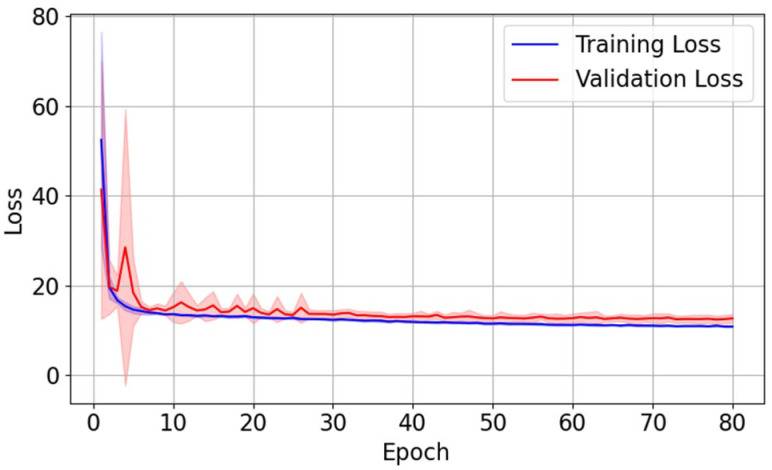
The average training (blue) and validation (red) loss during the training process, averaged over ten folds. The shades represent ± 1 standard deviation of training (shaded blue) loss and validation (shaded red) loss at different epochs in the ten-fold cross-validation.

The evolution of training loss and validation loss, averaged over ten folds, is shown in Figure 3. Both curves exhibit a sharp drop during early epochs of training, followed by a gradual plateau with minimal fluctuations, indicating stable convergence of training. The close alignment between the two curves suggests no obvious overfitting. 

**Figure 4:**
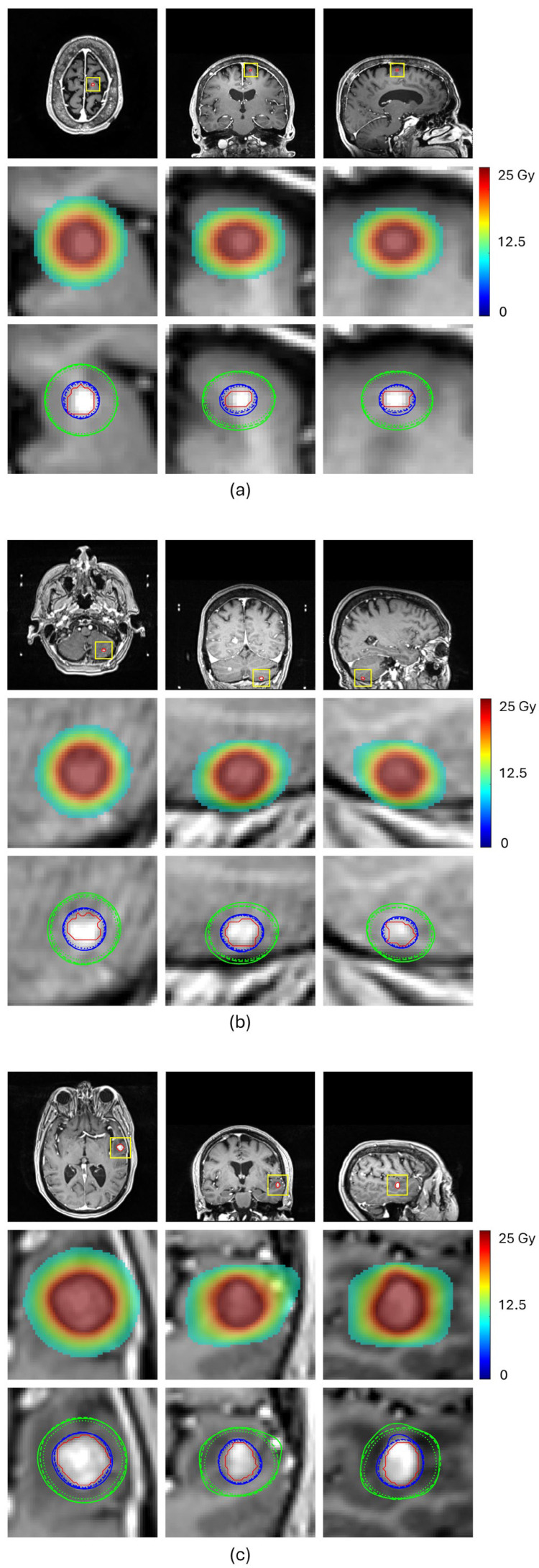

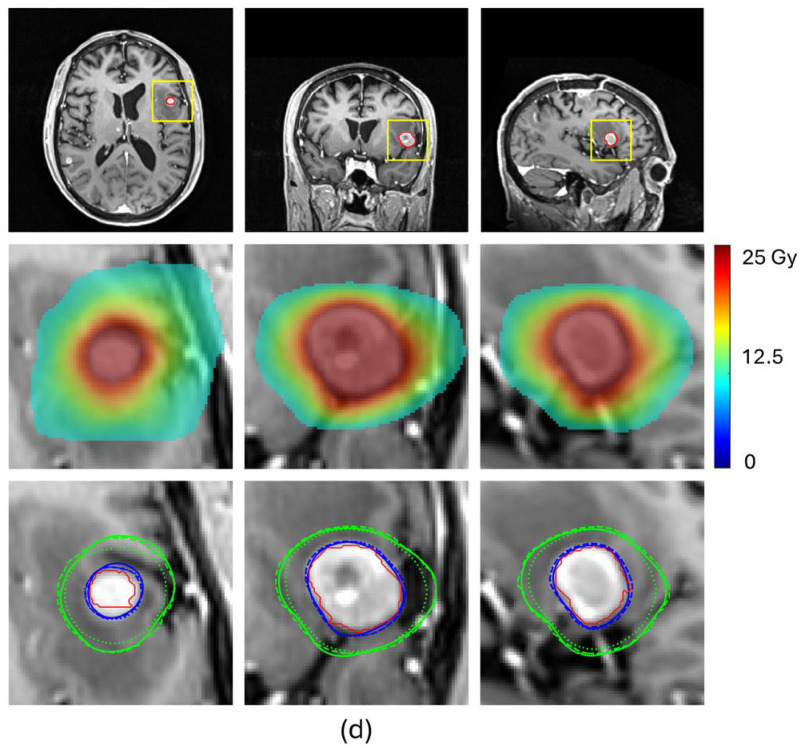
Subfigures (**a**–**d**) show the predicted results for the four representative BMs, respectively. In each subfigure, the first row displays MRI images in transverse, coronal and sagittal views, with the BM contour outlined in red; The second row presents a zoomed-in view of the BM region (as indicated by the rectangle in the first row), showing the ground truth dose distribution in color wash; The third row compares 100% isodose lines (yellow) and 50% isodose lines (green) from the ground truth dose distribution (solid lines), the dose predicted by the HD-U-Net trained with conventional MSE loss (dotted lines), and the dose predicted by the HD-U-Net model trained with our proposed method (dashed lines).

Table 2 and Figure 4 compare the prediction performance of our proposed method with a baseline HD-U-Net trained using the conventional MSE loss for four representative BMs, each selected from a different volume group. For the two small-volume BMs, BM 1 and BM2, our method achieved substantially higher DSC values for both 100% and 50% isodose lines (DSC_100%_: 0.908 vs. 0.790 for BM1 and 0.936 vs. 0.886 for BM2, DSC_50%_: 0.947 vs. 0.849 for BM1 and 0.908 vs. 0.827 for BM2), indicating markedly improved agreement with the ground truth dose distribution (Figure 4a,b). These gains translated into significant improvements in plan quality prediction. Selectivity error decreased from 0.19 to 0.06 for BM1 and from 0.11 to 0.06 for BM2, CI50 error reduced from −5.04 to −1.55 for BM1 and from −3.05 to −1.75 for BM2, and GI error from 0.91 to 0.56 for BM1 and from −0.53 to −0.27 for BM2. For the mid-volume BM3, while the baseline mode already produced reasonable similarity in the 100% isodose line, our method further improved the DSC value from 0.926 to 0.944, reducing selectivity error from 0.10 to 0.06. More notably, our method significantly increased the DSC value of the 50% isodose line from 0.847 to 0.925, consistent with the isodose lines shown in Figure 4c, reducing CI50 error from −1.25 to −0.40. With both isodose lines improved, the GI error decreased from −0.48 to −0.01. For the large-volume BM4, both methods achieved comparable similarity for the 100% isodose line (DSC_100%_: 0.952 vs. 0.951), while our method produced a higher DSC for the 50% isodose line (DSC_50%_: 0.951 vs. 0.912), as illustrated in Figure 4d. This improvement translated to a decrease in the CI50 error from −0.50 to 0.25 and the GI error from −0.63 to less than 0.01. These results demonstrate that our proposed method consistently enhances similarity of the 100% and 50% isodose lines of the predicted doses relative to those of ground truth doses, especially for small-volume lesions, leading to more accurate prediction of plan quality metrics. 

**Figure 5:**
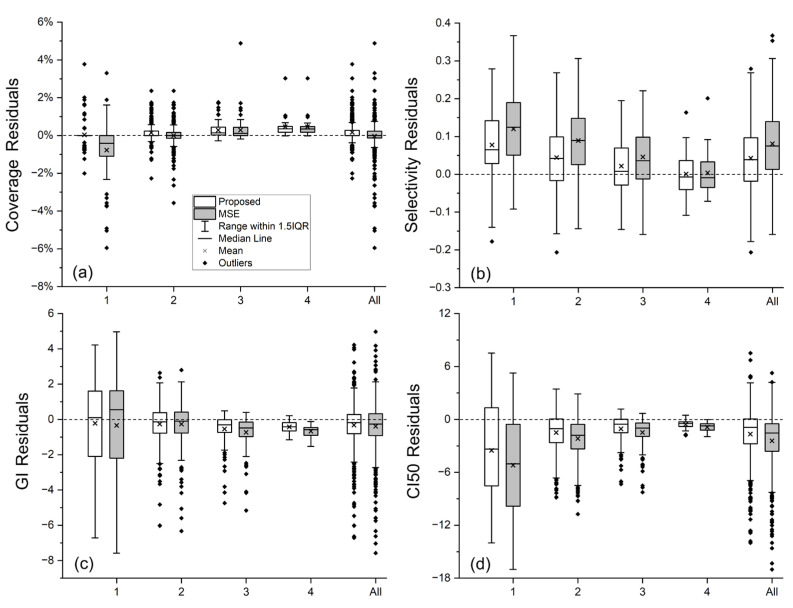
Box plots of prediction errors for four plan quality metrics: (**a**) coverage, (**b**) selectivity, (**c**) GI, and (**d**) CI50, across BM volume groups, comparing the proposed method with the baseline HD-U-Net model trained with conventional MSE loss. Boxes represent the 25%–75% interquartile range (IQR) of prediction errors, with the median shown as a solid line and the mean as a cross (×). Whiskers extend to 1.5 × IQR, and outliers (◆) are defined as cases with errors beyond the whiskers.

Table 3 summarizes the average values of coverage, selectivity, GI, and CI50 calculated from the ground truth dose distribution, the dose predicted by the baseline HD-U-Net model trained with conventional MSE loss, and the dose predicted by the HD-U-Net trained with our proposed method. Results are presented for each of the four BM volume groups and for the entire dataset. Table 4 presents the MAE values for each plan quality metric of both methods, while the box plots in Figure 5 complements Table 4 by showing distributions of residual errors for each metric prediction.

Given the consistently high coverage in the collected clinically approved plans (most BMs have coverage ≥ 99%), both the baseline and proposed methods predicted coverage with high accuracy, as reflected by the small MAE values in Table 4, except for group 1, the smallest-volume BMs. For these very small BMs, coverage is extremely sensitive, even a sub-voxel discrepancy in the 100% isodose line can noticeably alter the coverage value. By enforcing similarity on the 100% isodose line, our proposed method substantially improved coverage prediction accuracy in group 1, yielding more accurate average coverage value (99.79 ± 0.40 vs. 98.98 ± 1.42, ground truth: 99.75 ± 0.66), a relatively much smaller MAE value (0.38% vs. 1.09%), and shorter error bar representing 1.5 times the interquartile range (IQR), as shown in Figure 5.

For the other three plan quality metrics, our proposed method consistently produced average values closer to the ground truth (Table 3) and smaller MAE values (Table 4) than the baseline HD-U-Net trained with conventional MSE loss, demonstrating better agreement with ground truth in both overall trend and per-lesion accuracy. As illustrated in Figure 5, our method consistently outperformed the conventional MSE loss, achieving smaller mean and median prediction errors for all the three metrics in almost every volume group.

When comparing the results among volume groups, the largest improvements were seen in the two small-volume groups; For the mid- and large-volume groups, improvements are mainly observed in GI and CI50, which are the metrics directly related to the spatial extent of the 50% isodose line. This trend observed from Table 3 and Table 4 aligns with the prediction results of the four representative cases discussed earlier. Additionally, Figure 5 demonstrates that the improvements in the small-volume groups not only include reductions in the average prediction error for selectivity, GI, and CI50, but also include reduced prediction accuracy variability, as indicated by shorter bars representing 1.5IQR. The largest standard deviations of the ground truth plan quality metrics are observed in GI and CI50 for group 1 (Table 3), reflecting substantial quality variations in our collected plans for very small BMs. Because the network tends to capture dataset-level trends, such variations can result in larger prediction errors, which are consistent with the results shown in Table 4 and Figure 5. Notably, our proposed method was more robust to this variation than the conventional MSE loss, producing both more accurate average values of plan quality metric (trend-level agreement) and smaller MAE values (per-lesion accuracy).

We conducted paired Wilcoxon signed-rank tests to compare the prediction errors of our method with those of the baseline HD-U-Net model trained with conventional MSE loss. The two-sided *p*-values for this prediction error comparison are presented in Table 5. Figure 5 has illustrated that our method yielded smaller median prediction errors across all metrics for the smallest volume group; smaller median errors in selectivity and CI50 for the small-volume group 2; smaller median errors in selectivity, GI, and CI50 for the mid-volume group 3; and smaller median errors in GI and CI50 for the large-volume group 4. For the overall dataset, our method achieved smaller median errors in selectivity, GI, and CI50. The two-sided *p*-values in Table 5 complement these observations and confirm that these improvements achieved by our method are statistically significant (*p* < 0.05). We also compared the absolute prediction errors of the two methods, with one-sided *p*-values presented in Table 6. These results further demonstrate that our method achieved statistically significant reductions in median absolute prediction errors across all four metrics for the two small-volume groups (*p* < 0.05), as well as significant reductions in median absolute prediction errors in GI and CI50 for the mid- and large-volume groups (*p* < 0.05). For the overall dataset, our method yielded statistically significant reductions in median absolute errors in all four metrics (*p* < 0.05). 

## 4. Discussion

In this study, we developed a deep learning-based method for predicting per-lesion plan quality metrics for GK radiosurgery of BMs using a HD-U-Net model [13], with a particular emphasis on improving prediction accuracy across different lesion volumes. Given the substantial variability in the number, size, shape, and location of BMs across patients, we adopted a lesion-level network training strategy, as in Zhang et al. [23], rather than the more common patient-level approach used in studies of primary cancers [12,13,14,16,17]. In addition, because the GK plan quality metrics are derived from the 100% and 50% isodose lines of the plan dose distribution, we incorporated a DSC loss to the conventional MSE loss to explicitly enforce similarity on these two clinically critical isodose lines of the predicted dose relative to those of the ground truth dose, aiming to improve the prediction accuracy of the plan quality metrics. Compared with the baseline HD-U-Net model trained with conventional MSE loss, our proposed approach consistently achieved more accurate average values of plan quality metrics with lower MAE across different BM volume groups. 

One of the most notable findings was the performance gain for the smallest-volume BMs (group 1). Due to the extreme sensitivity of coverage in these cases, where even sub-voxel discrepancy in the 100% isodose line can substantially alter the metric value, accurate prediction is challenging. The marked improvements of our method in coverage prediction for these small BMs suggest that incorporating structural constraints on clinically critical isodose line helps overcome this challenge. In addition, much larger deviations in ground truth GI and CI50 values were also found for group 1, reflecting substantial variability in treatment planning for these small BMs. As deep learning models tend to learn dataset-level trend, such variability can limit per-lesion accuracy. Nevertheless, our approach demonstrates improved robustness to these data variations, achieving both better agreement in overall trend and higher per-lesion accuracy. For mid- and large-volume BMs, decent improvements were observed primarily in GI and CI50, both of which are directly related to the spatial extent of the 50% isodose line. The consistent reduction in prediction errors for these metrics with statistical significance indicates that our method better captures the dose fall-off characteristics in the collected GK plans. Accurate prediction of these metrics is important for balancing target coverage with normal brain tissue sparing, which is a critical consideration in GK radiosurgery. 

As described in Section 2.1, our definition of the tumor space as the 30% isodose volume reflects a practical balance between capturing a sufficiently large dose region and maintaining computational efficiency. In future work, we plan to conduct a comprehensive study to assess the sensitivity of model performance to this choice. The weights in the proposed loss function were determined through empirical hyperparameter tuning with a coarse logarithmic grid search. This study serves as a proof of concept to demonstrate the benefit of enforcing similarity on clinically relevant isodose lines during network training. Moving forward, we plan to perform systematic sensitivity analyses and optimize hyperparameter values to further enhance model performance.

This study has two limitations. First, BMs located near critical organs or adjacent to other BMs were excluded due to their limited number in our dataset and their distinct dose distribution patterns caused by OAR sparing or by the interplay with dose contributions from neighboring BMs. For BMs near critical organs, OAR sparing needs to be considered during treatment planning, which alters the resulting dose distribution. The required extent of OAR sparing varies across cases, depending on factors such as the lesion-organ spatial relationship, prescription dose, organ tolerance, and dose contributions from other BMs near the same organ, if present. Learning this complex interplay with deep learning would require a large number of such BMs; otherwise, the model would risk significant overfitting. For BMs whose dose distributions overlap with those of adjacent lesions, their plan quality metrics such as selectivity, GI, and CI50 cannot be calculated and are reported as unavailable in the Leksell Gamma Plan system. We excluded these BMs to ensure that the dose distribution of each BM included in our dataset could be analyzed independently. Moreover, the final composite dose distribution of adjacent BMs depends on their prescription doses, spatial relationships, and potential overlaps with critical organs. Learning such highly case-specific interactions would also require a substantially larger dataset to avoid overfitting. In future work, we will continue collecting GK plans from new patients and incorporate these types of BMs into the training dataset once sufficient numbers are available. 

Second, although the collected GK plans were clinically approved for patient treatments, they represent clinically acceptable plan quality but not necessarily the best achievable quality for each individual patient, and quality variations exist across these plans. While our method has shown improved robustness to such variability, it still inherently limits per-lesion prediction accuracy. Moreover, since the ground truth metrics for the testing cases were from these clinical plans, the inherent variability of these metrics also inevitably constrains the accuracy of per-lesion evaluation. To account for this limitation, we illustrated per-lesion prediction errors using box plots to show the interquartile range and employed paired Wilcoxon signed-rank tests, both of which provide some robustness against outliers.

Despite these limitations, our proposed plan quality prediction network is expected to provide a reference plan quality that represents the average or majority level of clinically accepted quality achieved in prior cases with similar anatomies. It can be integrated into routine GK planning workflow in two ways. One is as a pre-planning tool to predict a reference plan quality in advance, offering some guidance during manual priority tuning of inverse planning. Although BMs near critical organs were excluded from training, the network can be used to predict the achievable dose distributions for all BMs when OAR sparing is not considered. By defining the tumor space as the 30% isodose volume for training, we ensured that even at the highest prescription dose, the 30% isodose level remains below the lowest cranial OAR tolerance. Thus, for BMs near critical organs, the predicted dose distribution within the 30% isodose level can still serve as a useful reference, enabling planners to pre-evaluate the need and extent of OAR sparing and inform appropriate dose constraints. Similarly, although trained on non-adjacent BMs, we can use our network to predict dose distributions for all BMs when contributions from neighboring lesions are not considered. Because the Leksell Gamma Plan system only supports independent, sequential planning for each BM, planners must iteratively evaluate composite dose distribution and adjust each BM plan when lesions are close to one another. Summing predicted dose distributions of all BMs allows pre-evaluation of potential dose contributions from neighboring BMs, which can help guide manual priority tuning for each BM plan and potentially reduce the number of iterative plan adjustments and composite dose evaluations. In future work, we plan to see IRB approval to prospectively evaluate this pre-planning application. Specifically, in each new case, one experienced planner will be provided with predicted plan quality prior to planning, while another experienced planner will follow the standard workflow without access to predictions. The resulting plans will be compared using paired statistical tests to determine whether access to predictions leads to statistically better plans. 

Another way to integrate our prediction network into routine GK planning workflows is as a post-planning quality control tool. After plan generation, planners can compare the obtained plan quality with the predicted reference quality. If the current quality falls below the predicted quality by more than a specified threshold, the planner will further refine the plan. Given the current limitation in per-lesion prediction accuracy due to variability in the training data, we envision initially using relatively loose actionable criteria to flag only those plans that fall substantially below the predicted quality. In the long term, using our network as a quality control tool in an iterative process could gradually mitigate this limitation. By identifying and refining plans that fall below the predicted quality beyond the specified threshold, overall plan quality and consistency would improve, which would in turn provide an enhanced dataset to retrain the model. Over multiple iterative rounds, we anticipate this process to progressively enhance the quality of the training data and improve the model’s performance not only in per-lesion accuracy but also in its ability to predict optimal achievable plan quality. As model performance improves across iterations, progressively stricter actionable criteria could be adopted. The optimal actionable criteria at each stage can be determined through prospective studies. For example, for each new case, a second experienced planner could attempt to refine the plan generated by another planner, without access to the reference quality predicted by our network. Given a specified actionable criterion, the performance of our network for quality control can be categorized for each BM as: (1) true negative, if the current plan is comparable to or better than the predicted quality, and the refinement does not yield any improvement; (2) false negative, if the current plan is comparable to or better than the predicted quality, but refinement yields improvement; (3) true positive, if the current plan is worse than the predicted quality, and refinement improves it; (4) false positive., if the current plan is worse than the predicted quality, but refinement does not yield any improvement. Based on these outcomes, we can calculate sensitivity and specificity. The optimal actionable criterion will be selected to balance sensitivity and specificity, depending on whether the clinical priority is to maximize detection of suboptimal plans or to reduce inefficiency from excessive unnecessary refinement attempts. 

Beyond these two direct clinical applications, more reliable plan quality predictions across lesion sizes can also facilitate the development of DRL-based automatic planning by providing lesion-specific reference quality benchmarks for reward function design.

## 5. Conclusions

We have developed and validated a deep learning-based approach for per-lesion plan quality prediction in GK radiosurgery for BM patients. By incorporating a DSC loss term to the conventional MSE loss to enforce similarity on clinically critical isodose lines during network training, our method outperformed the MSE loss in capturing overall trends, improving per-lesion prediction accuracy, and enhancing robustness to dataset variability. This framework has the potential to be integrated into clinical practice either as a pre-planning tool to guide manual priority tuning during inverse planning, or as a post-planning quality control tool to identify and refine plans that fall below predicted quality, thereby improving quality consistency across cases. In addition, more reliable patient-specific plan quality prediction can support the development of DRL-based automated planning for GK radiosurgery of BMs by informing reward function design, maintaining consistently high plan quality, and reducing inter-planner variability.

## Figures and Tables

**Table 1:** Detailed information on our final dataset.

Number of patient cases	175
Number of BMs included	463
BM volume (mm^3^)	14.875–5995.875
Prescription dose (Gy)	21, 20, 18, 17, 16, 15, 14, 12
Coverage (%)	95.0–100.0
Selectivity	0.188–0.897
GI	2.467–16.761
CI50	2.667–31.607

**Table 2:** Prediction results for four representative BMs, each selected from a different BM volume group. For each case, plan quality metrics were calculated for the ground truth dose distribution, the dose predicted by the HD-U-Net network trained with conventional MSE loss, and the dose predicted by the HD-U-Net network trained with our proposed method, respectively. The DSC values of the 100% and 50% isodose lines between the ground truth and predicted dose distributions are also computed, denoted as DSC_100%_ and DSC_50%_, respectively.

ID	BM Volume (mm^3^)	Method	DSC_100%_	DSC_50%_	Coverage (%)	Selectivity	GI	CI50
BM1	20.5	Ground truth	NA	NA	100	0.37	7.09	19.18
		MSE	0.790	0.849	99.39	0.56	8.00	14.14
		Proposed	0.908	0.947	100	0.43	7.65	17.63
BM2	74.5	Ground truth	NA	NA	100	0.44	4.53	10.34
		MSE	0.886	0.827	99.66	0.55	4.00	7.29
		Proposed	0.936	0.908	100	0.50	4.26	8.59
BM3	536.5	Ground truth	NA	NA	99.98	0.66	3.16	4.78
		MSE	0.926	0.847	100	0.76	2.68	3.53
		Proposed	0.944	0.925	100	0.72	3.15	4.38
BM4	3164.0	Ground truth	NA	NA	99.59	0.88	2.78	3.13
		MSE	0.951	0.912	99.98	0.81	2.15	2.63
		Proposed	0.952	0.951	99.98	0.82	2.78	3.38

**Table 3:** Average values of coverage, selectivity, GI, and CI50 calculated from the ground truth dose distribution, the dose predicted by the baseline HD-U-Net model trained using the conventional MSE loss, and the dose predicted by the HD-U-Net trained with our proposed method. Average plan quality metrics are also reported separately for the four BM volume groups.

Metric	Method	BM Volume Group				
		Group 1	Group 2	Group 3	Group 4	All
Coverage (%)	Ground truth	99.75 ± 0.66	99.77 ± 0.42	99.67 ± 0.58	99.52 ± 0.60	99.73 ± 0.51
	MSE	98.98 ± 1.42	99.77 ± 0.47	99.95 ± 0.07	99.98 ± 0.03	99.70 ± 0.73
	Proposed	99.79 ± 0.40	99.92 ± 0.28	99.95 ± 0.33	99.99 ± 0.01	99.91 ± 0.31
Selectivity	Ground truth	0.41 ± 0.09	0.49 ± 0.11	0.67 ± 0.10	0.79 ± 0.07	0.53 ± 0.14
	MSE	0.54 ± 0.07	0.58 ± 0.06	0.71 ± 0.07	0.79 ± 0.03	0.61 ± 0.10
	Proposed	0.49 ± 0.06	0.53 ± 0.06	0.69 ± 0.07	0.79 ± 0.03	0.57 ± 0.10
GI	Ground truth	7.83 ± 2.91	4.55 ± 1.48	3.47 ± 0.97	2.81 ± 0.28	4.74 ± 2.18
	MSE	7.49 ± 1.44	4.29 ± 1.01	2.75 ± 0.29	2.14 ± 0.22	4.35 ± 1.80
	Proposed	7.61 ± 1.26	4.28 ± 0.89	2.93 ± 0.19	2.39 ± 0.20	4.42 ± 1.72
CI50	Ground truth	19.02 ± 5.80	9.70 ± 3.56	5.38 ± 1.96	3.57 ± 0.58	9.91 ± 5.70
	MSE	13.82 ± 2.06	7.51 ± 2.07	3.90 ± 0.66	2.69 ± 0.30	7.48 ± 3.65
	Proposed	15.51 ± 2.50	8.20 ± 2.27	4.32 ± 0.64	3.02 ± 0.26	8.25 ± 4.10

**Table 4:** Mean absolute errors (MAEs) for plan quality metrics predicted by our proposed method relative to ground truth values. Calculations were performed within each volume group and for the entire dataset. For comparison, the MAE was also computed for metrics predicted by the baseline HD-U-Net model trained with conventional MSE loss.

MAE	Method	BM Volume Group				
		Group 1	Group 2	Group 3	Group 4	All
Coverage (%)	MSE	1.09 ± 1.39	0.33 ± 0.50	0.31 ± 0.57	0.46 ± 0.61	0.45 ± 0.77
	Proposed	0.38 ± 0.68	0.26 ± 0.42	0.29 ± 0.37	0.47 ± 0.60	0.29 ± 0.47
Selectivity	MSE	0.13 ± 0.09	0.10 ± 0.07	0.07 ± 0.05	0.05 ± 0.04	0.10 ± 0.07
	Proposed	0.09 ± 0.07	0.08 ± 0.06	0.06 ± 0.05	0.05 ± 0.04	0.07 ± 0.06
GI	MSE	2.23 ±1.71	0.84 ± 0.88	0.79 ± 0.93	0.67 ± 0.33	1.04 ± 1.16
	Proposed	1.95 ± 1.48	0.80 ± 0.86	0.63 ± 0.87	0.44 ± 0.30	0.92 ± 1.07
CI50	MSE	6.21 ± 4.51	2.43 ± 2.14	1.58 ± 1.74	0.88 ± 0.51	2.75 ± 2.96
	Proposed	5.28 ± 3.89	2.00 ± 1.91	1.25 ± 1.62	0.65 ± 0.47	2.28 ± 2.60

**Table 5:** Two-sided *p*-values from paired Wilcoxon signed-rank tests, comparing the prediction errors of plan quality metrics between our proposed method and the baseline HD-U-Net model trained with conventional MSE loss. The tests were conducted for each volume group and for the entire dataset.

Two-Sided *p*-Values	BM Volume Group				
	Group 1	Group 2	Group 3	Group 4	All
Coverage	<0.05	<0.05	<0.05	0.21	<0.05
Selectivity	<0.05	<0.05	<0.05	0.31	<0.05
GI	<0.05	0.70	<0.05	<0.05	<0.05
CI50	<0.05	<0.05	<0.05	<0.05	<0.05

**Table 6:** One-sided *p*-values from paired Wilcoxon signed-rank tests, comparing the absolute prediction errors of plan quality metrics between our proposed method and the baseline HD-U-Net model trained with conventional MSE loss. The tests were conducted for each volume group and for the entire dataset.

One-Sided *p*-Values	BM Volume Group				
	Group 1	Group 2	Group 3	Group 4	All
Coverage	< 0.05	< 0.05	0.11	0.12	< 0.05
Selectivity	< 0.05	< 0.05	0.09	0.42	< 0.05
GI	< 0.05	< 0.05	< 0.05	< 0.05	< 0.05
CI50	< 0.05	< 0.05	< 0.05	< 0.05	< 0.05

## Data Availability

Restrictions apply to the availability of these data. Data were obtained from Emory University and are available from Dr. Xiaofeng Yang upon reasonable request, subject to approval by Emory University IRB.

## Abbreviations

The following abbreviations are used in this manuscript:

GK	Gamma Knife
BM	Brain metastasis
GAN	Generative adversarial network
HD-U-Net	Hierarchically densely connected U-Net
DSC	Dice similarity coefficient
MSE	Mean squared error
IRB	Institutional Review Board
GI	Gradient index
CI50	Conformity index at 50% of prescription dose
ReLU	Rectified linear unit
DRL	Deep reinforcement learning
IQR	Interquartile range
OAR	Organs at risk
